# Effect of apical microsurgery on force regulation of incisor teeth during unpredictable force control task

**DOI:** 10.1111/joor.13334

**Published:** 2022-05-16

**Authors:** Khaled Al‐Manei, Nabeel Almotairy, Kholod Khalil Al‐Manei, Anastasios Grigoriadis, Abhishek Kumar

**Affiliations:** ^1^ Division of Oral Diagnostics and Rehabilitation Department of Dental Medicine Karolinska Institutet Huddinge Sweden; ^2^ Division of Endodontics Department of Restorative Dental Science College of Dentistry King Saud University Riyadh Saudi Arabia; ^3^ Department of Orthodontics and Pediatric Dentistry College of Dentistry Qassim University Buraidah Saudi Arabia

**Keywords:** apical surgery, force control, force rate, incisors, tooth apex

## Abstract

**Background:**

Apical microsurgery (AMS) involves removal of the root‐end which can affect the force regulation of teeth.

**Objective:**

To investigate the force regulation of incisor teeth treated with AMS during the unpredictable force control task in comparison with their contralateral teeth with complete root apices, in humans.

**Methods:**

Fifteen eligible participants (8 women and 7 men; mean age 52.9 ± SD 4.4 years) performed a standardised unpredictable force control task, which involved pulling and holding a force transducer with AMS‐treated incisors and their contralateral control teeth (*n* = 30 teeth). A series of four load masses: 100, 200, 50 and 300 gm were attached to the force transducer through a string in an unpredictable manner. The obtained force profile was divided into initial and later time‐segments. The peak force and peak force rate during the initial time‐segment, and the holding force and coefficient of variability during the later time‐segments were calculated and compared by the repeated measures analysis of variance.

**Results:**

During the initial time‐segment, the peak force and peak force rate were significantly lower in the AMS‐treated teeth than in the controls (*p* = .001, *p* = .013, respectively). However, during the later time‐segment, no significant differences in the holding force nor the coefficient of variability were observed between the AMS‐treated teeth and their controls (*p* = .755, *p* = .213, respectively).

**Conclusion:**

In contrast to incisors with complete normal root apices, AMS‐treated incisors do not show robust changes in force regulation.

## Graphical abstract

During an unanticipated force control task, incisor teeth treated with apical microsurgery (AMS) demonstrate similar force regulation to natural teeth with complete normal root apices. Aside from the healing of post‐treatment apical periodontitis, AMS treatment plays a crucial role in maintaining tooth function.

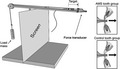

## 1 INTRODUCTION

Apical surgery is usually considered as a last resort over tooth extraction to preserve an endodontically treated tooth with post‐treatment apical periodontitis.^1^, ^2^, ^3^ Over time, apical surgery has endured many advances and turned into microsurgical procedures, in which visualisation of the root‐end structures using the dental surgical microscope becomes easier for the clinicians.^4^ These advances have resulted in the ability to perform apical microsurgery (AMS) with a high degree of precision, leading to a more favourable treatment outcome.^5^

The force regulation of teeth is determined by the ability of afferent neurons in the sockets to signal the load and encode the information direction of the applied force/load.^6^ Sensory information plays a critical role in the regulation of forces and movements necessary for oral motor activities such as biting and chewing.^7^, ^8^, ^9^, ^10^ Previous studies have shown an impaired force control due to disruption or disturbance of such sensory information from the afferent neurons that surrounded the teeth roots.^11^, ^12^, ^13^, ^14^ The impaired force control was evident in the higher ‘holding’ forces in a standardised biting task.^13^, ^15^, ^16^ Therefore, unperturbed oral movements and oral fine motor control depend on the ability of the central nervous system to process the sensory information and regulate the forces needed to execute the function.

When performing a specific motor function, efficient motor commands are generated for achieving the desired outcome.^17^ These commands depend on the motor system's ability to predict the outcome based on the sensory information.^17^ The prediction is also reliant on the sensorimotor memory and previous experience that are regarded as crucial elements to develop the appropriate motor commands to facilitate efficient function.^18^ A loss or disturbance of sensory information may compromise the predictability and eventually impair the force regulation in the motor tasks. Previous studies have investigated the effect of unpredictable load demands on motor control strategies in children and adults.^8^, ^9^ However, there is limited information on motor control strategies and force regulation during unpredictable load demands in endodontically treated teeth.^7^

The goals of AMS treatment are not only restricted to the absence of signs and symptoms or resolution of apical periodontitis,^5^ but also extended to preservation of tooth function and patient satisfaction.^19^, ^20^ Previous studies have shown that the majority of the neural afferents that convey information about occlusal forces are found in the apical third of the periodontal ligament near the root apex.^21^ AMS treatment typically involves the removal of 2–3 mm of the root apex and the surrounding periapical lesions.^4^ Therefore, it can be hypothesised that this procedure may perturb the sensory signals from dental afferents to the brain, affecting the force regulation of the teeth and failure to achieve the objective of the oral motor task such as biting. Previous studies have also shown that any perturbation in the neuronal signals would be reflected in increased forces during the force control task.^12^, ^14^, ^22^, ^23^ Thus, the purpose of the current exploratory pilot study was to investigate the force regulation of incisor teeth treated with AMS during the unpredictable force control task in comparison with their contralateral control teeth with complete root apices, in humans. We hypothesised that perturbation in the sensory inputs due to resection of root apex during AMS will result in higher holding forces during the unpredictable force control task compared with the control teeth.

## 2 METHODS

### 2.1 Ethical consideration

The study protocol was approved by the Regional Ethical Review Board in Stockholm, Sweden (Dnr: 2018/1963‐31) and conducted in accordance with the Declaration of Helsinki. This study also complies with the ‘strengthening the reporting of observational studies in Epidemiology’ (STROBE) checklist.

### 2.2 Participants’ selection

Patients (eight women and seven men; mean age 52.9 ± standard deviation 4.4 years old) who were treated with AMS on their upper incisors’ teeth between January 2013 and December 2018 at the Endodontic Specialist Clinics, Department of Dental Medicine, Karolinska Institutet, Sweden, were identified from the electronic dental records. AMS treatment was performed following the contemporary AMS protocol using mineral trioxide aggregate (MTA) as a retrograde filling material.^1^ Seventeen patients met the inclusion criteria, and 15 of them agreed to participate in the study. The inclusion criteria were as follows: a recall periapical radiograph demonstrating complete healing^24^, ^25^ with a minimum of one‐year follow‐up, an adequate coronal restoration, a crown‐to‐root ratio is ≤1:1, and the presence of an antagonist's tooth (controls; diagnosed with normal pulp and normal periapical tissues) with no history of apical surgery. Exclusion criteria were self‐reports of marked systemic, neurological, infectious diseases or painful conditions, temporomandibular disorders, history of orthodontic treatment, periodontal diseases, teeth mobility and moderate‐to‐severe malocclusion. All 15 participants signed a written informed consent before commencing the experiment.

### 2.3 Armamentarium and experimental protocol

**FIGURE 1:**
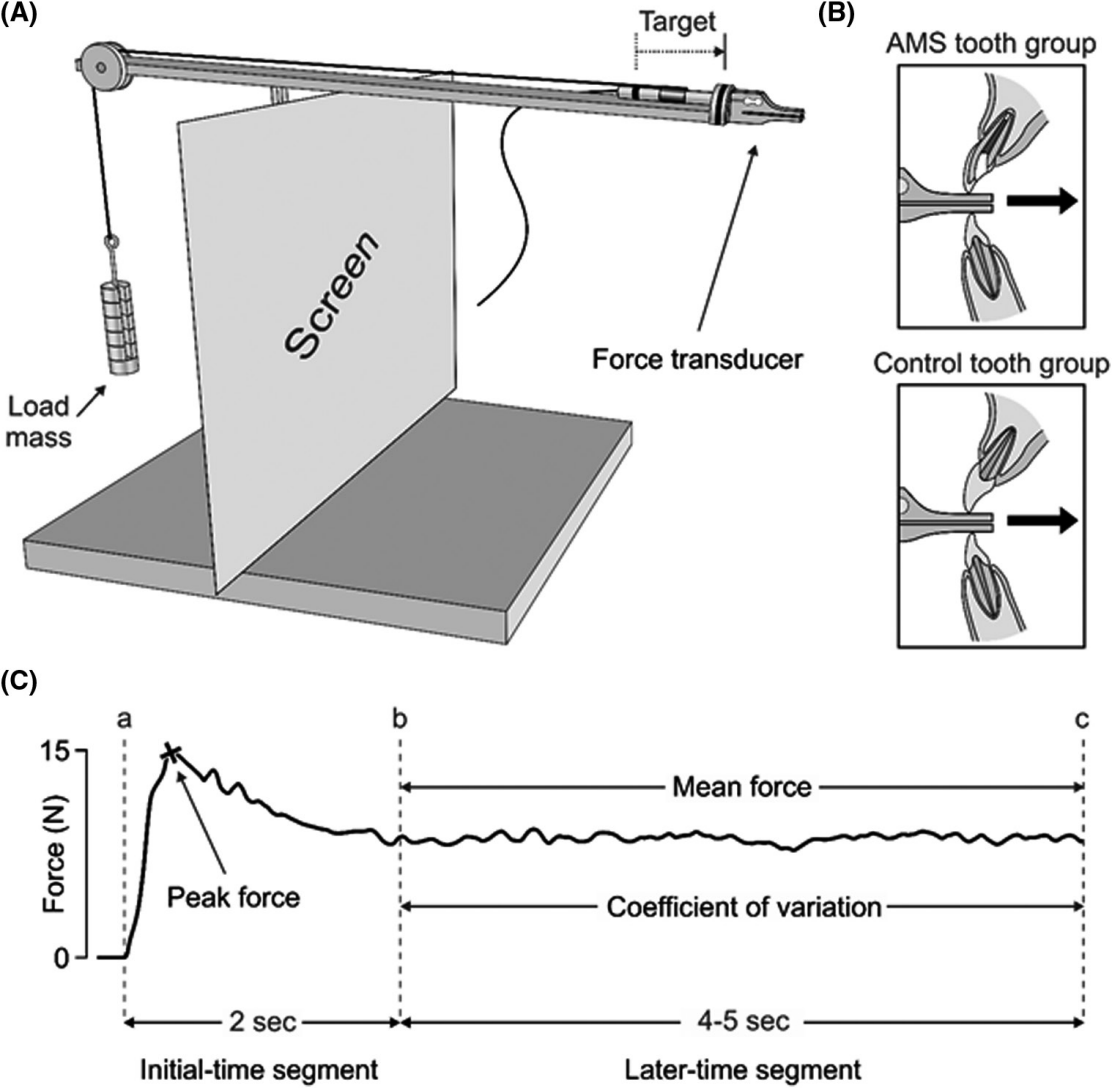
(A) Schematic illustration of the task apparatus which consists of a force transducer resting on a platform and tied to a string. The string passes through a pulley and terminated in a metal hook to which different four load masses (100, 200, 50 and 300 gm) could be attached. (B) A close‐up view of AMS and control teeth groups, where each participant was asked to pull the force transduce to the targeted black line. (C) An example of a temporal force profile in Newton was obtained from a single trial for a tooth with AMS. The entire force profile lasted for 6–7 s and was divided into initial and later time‐segments. The initial time‐segment represents the first 2 s of the force profile (lines a–b), whereas the late time‐segment represents the remained force profile (lines b‐c). The peak force and peak force rate variables were calculated during the initial time‐segment, while the mean holding force and the coefficient variability of holding force were calculated during the later time‐segment

The volunteers participated in a single experimental session, where they performed the unpredictable force control task involving the upper incisor teeth (Figure 1A). The detailed description of the task and the specifications of the apparatus are described in the previous studies.^8^, ^9^ In brief, the apparatus consisted of a custom‐built strain gauge‐based force transducer (Department of Integrative Medical Biology, Umeå University,) resting on a platform and tied with a string (0.25 mm diameter and 5 kg strength; Master Line Kayoba; Jula,). The string passes through a pulley and is terminated in a metal hook, where different standardised metallic loads (Viktsats; Sagitta Pedagog AB,) could be attached. A sequence of four load masses was attached to the metallic hook in random order: 100, 200, 50 and 300 gm. Each load mass was repeated 3 times before changing to the next load mass in the sequence, rendering a total of 12 trials per tested tooth and 24 trials in total during the experiment session.

### 2.4 The unpredictable force control task

The participants sat on an office chair with arm support in a quiet room. The task apparatus was placed on an adjustable table in front of each participant to ensure a comfortable biting position during the task. Two black lines were drawn on the apparatus (5.5 cm apart). One line was drawn on the terminal end of the platform (where the force transducer is supported). Another line was drawn on the force transducer. An independent ‘blinded’ trained examiner (NA) explained the unpredictable force control task to the participants. Accordingly, each participant was instructed to bite and gradually pull the force transducer by their upper incisor tooth treated with AMS or its contralateral control and attempt to match the two black lines (Figure 1B). Once the two lines are matched, the participant was asked to hold the transducer for 4–5 s (s) before returning it to its initial position. For each tooth, the participant performed a familiarisation session of 5 trials of biting and pulling 50 gm load mass. After the familiarisation session, the participants performed the actual task using the AMS‐treated tooth and its control individually. The order of the teeth (AMS/contralateral control) and the sequence of load (50/100/200/300) were randomised and pre‐generated with a web‐based random order code (www.random.org). Before each biting and pulling attempt, the load masses were changed regularly in the above‐mentioned load sequence behind a screen and obscured from the view of the participants.

### 2.5 Data processing and analysing

The unpredictable force control task's force profile was sampled at 1000 Hz (low pass filtered 250 Hz) and then assessed using a laboratory computer system (WinSC/WinZoom, Department of Integrative Medical Biology, Umeå University,). Figure 1C presents the temporal force profile that was obtained from one trial for a tooth treated with AMS. The complete force profile took approximately 6–7 s and was divided into an initial time‐segment indicating the force profile overshoot's first 2 s (lines a‐b, Figure 1C) and a later time‐segment indicating the force profile's stabilised 4–5 s (lines b‐c, Figure 1C). To detect these two time‐segments, computer software was used, and their accuracy was checked manually. Further, the initial time‐segment's outcome variables were the peak force and the peak force rate measured in Newton (N) and Newton per second (N/s), respectively. The later time‐segment's outcome variables were the mean holding force (N) and the coefficient of variability of the holding force; intra‐trial force variability (%). The peak force rate was obtained by dividing the peak force over the elapsed duration to reach the peak (N/s). For calculating the CV, the force's standard deviation during the later time‐segment was divided over the mean force of the same period.^8^, ^9^

### 2.6 Statistical analysis

The normality of the data for each outcome variable (i.e. the peak force, peak force rate, holding force and coefficient of variability of the holding force) was tested using the Shapiro–Wilk test and histogram plots. The data appeared to be right‐skewed; therefore, the variables were logarithmically transformed. The logarithmically transformed data were then analysed by 2 × 4 repeated measures analysis of variance (ANOVA) models. The first factor in the ANOVA test was the teeth groups (two levels: control teeth versus the AMS‐treated teeth), and the second factor was the load masses (four levels: 50, 100, 200 and 300 gm). If the ANOVA models revealed a statically significant main effect and/or significant interactions (*p* < .05), a Tukey's honestly post hoc test was applied.

## 3 RESULTS

All participants performed the unpredictable force control task using the AMS‐treated teeth and their controls with no apparent difficulties. Below is a summary of the outcomes variables results between the AMS‐treated teeth and the controls (Table 1).

**TABLE 1:** Summary of the statistical results obtained from the repeated measures analysis of variance (ANOVA) two‐way model

Outcome variables	Teeth group (main effect)	Load masses (main effect)	Teeth group ×Load masses (interaction)
Peak force	*p* = .015*	*p* = .001*	*p* = .133
Peak force rate	*p* = .043*	*p* = .041*	*p* = .013*
Holding force	*p* = .062	*p* = .0001*	*p* = .755
Coefficient of variability	*p* = .366	*p* = .016*	*p* = .213

*Statistical significance (*p* < .5).

### 3.1 Peak force

**FIGURE 2:**
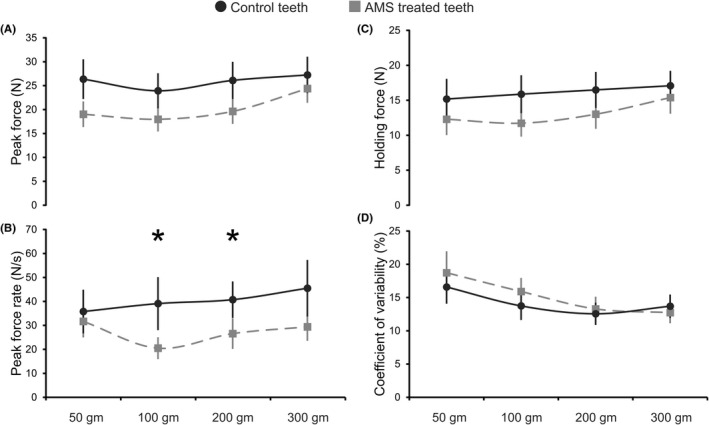
Line graphs representing the means (horizontal lines) and the standard deviations (vertical lines) of each study outcome variable prevailed by the control and AMS‐treated teeth during the unpredictable force control task. (A) The peak force, (B) the peak force rate, (C) the holding force and (D) the coefficient variability of the holding force for the control and AMS‐treated teeth using four load masses (50, 100, 200 and 300 gm). (*) Denotes significant differences between the teeth groups and the load masses in the interaction of study outcome variables

The mean values and the standard deviations of the peak force for the AMS and the control teeth are illustrated in Figure 2A. The peak force during the initial time‐segment of the force profile revealed a statistically significant main effect of the teeth groups (*F*_1,14_ = 7.619, *p* = .015). Particularly, teeth treated with AMS showed lower peak force compared with the control teeth. A significant main effect of load masses on the peak force was also observed (*F*_3,42_ = 9.423, *p* = .001). Post hoc testing of this main effect showed that the peak forces were significantly higher for 300 gm load mass in comparison with 50, 100 and 200 load masses (*p* < .017). Overall, there was no significant interaction of the peak force between the teeth groups and load masses (*F*_3,42_ = 1.972, *p* = .133).

### 3.2 Peak force rate

The mean values and the standard deviations of the peak force rate for the AMS and the control teeth are presented in Figure 2B. The peak force rate during the initial time‐segment exhibited a statistically significant main effect of teeth groups (*F*_1.14_ = 4.922, *p* = .043), indicating a lower peak force rate in teeth treated with AMS as compared to the control. A statistically significant main effect of the load masses on the peak force rate was also observed (*F*_3,42_ = 2.984, *p* = .041). Post hoc testing of this main effect showed that the peak force rates were only significant between 100 and 300 gm load masses (*p* = .026). Moreover, a significant interaction of the peak force rate was observed between the teeth groups and the load masses’ main effects (*F*_3,42_ = 4.059, *p* = .013). Post hoc analyses of this interaction showed a significantly lower peak force rate for the teeth treated with AMS than the control for 100 and 200 gm load masses (*p* < .005; respectively).

### 3.3 Holding force

The mean values and the standard deviations of the holding force for the AMS and the control teeth are illustrated in Figure 2C. The holding force during the later time‐segment of the force profile revealed no significant main effect of teeth groups (*F*_1,14_ = 4.11, *p* = .062), but a significant main effect of load masses was detected (*F*_3,42_ = 9.353, *p* = .0001). Post hoc testing of this main effect showed that the holding forces were significantly higher for 300 gm load mass in comparison with 50 and 100 gm load masses (*p* < .001). However, no significant interaction of the holding force was observed between the teeth groups and the load masses’ main effects (*F*_3,42_ = 0.39796, *p* = .755).

### 3.4 Coefficient of holding force variability

The mean values and the standard deviations of the coefficient variability of the holding force for the AMS and the control teeth are presented in Figure 2D. The coefficient of variability of the holding force during the later time‐segment exhibited no significant main effect of teeth groups (*F*_1,14_ = 0.872, *p* = .366), but a significant main effect of load masses was observed (*F*_3,42_ = 3.869, *p* = .016). Post hoc testing of this main effect showed that the coefficient of variabilities in the holding forces was significantly lower for 50 gm load mass in comparison with 200 and 300 gm load masses (*p* = .023, *p* = .035; respectively). Overall, there was no significant interaction in the coefficient of variability of the holding force between the teeth groups and the load masses’ main effects (*F*_3,42_ = 1.562, *p* = .213).

## 4 DISCUSSION

The force regulation necessary for oral motor control relies on the capacity of the central nervous system to collect, control and interpret the sensory information related to the task.^26^ Errors in processing sensory information can result in discrepancies in developing a suitable motor programme as well as failure to attain the task objectives.^27^ Previous studies have assessed oral motor control in participants who have healthy teeth from various age groups during unpredictable force tasks.^8^, ^9^ These studies observed that the oral motor control and force regulation for children and adults are similar. There are no studies, however, that have examined how different dental interventions impact oral motor control during unpredictable force control tasks. The present study thus examined the force regulation of incisor teeth that are treated with AMS in comparison with their contralateral control teeth during the standardised unpredictable force control task that involved pulling and holding different load masses. According to the findings, a considerably lower peak force and force rate were observed for AMS‐treated teeth compared with the control teeth in terms of the force profile's initial time‐segment. On the contrary, the holding force and the coefficient variability of the holding force did not show any major differences between the AMS‐treated teeth and their controls in terms of the force profile's later time‐segment. Despite the loss of the root‐end from the incisor teeth that were treated with AMS, the findings from the current study show no robust changes in force regulation compared with incisors with complete normal root apices.

The anticipation of the load masses during the unpredictable force control task has a crucial role in modifying the motor programme in accordance with the obtained sensory information.^8^, ^9^ The applied forces’ relationship in terms of the load masses follows Henneman's size principle of motor neuron activation wherein smaller low‐force neurons are first activated and then larger high‐force neurons follow.^28^ It is suggested that this orderly recruitment of the motor neurons will optimise the force control during the unpredictable force control task. Similarly, in the current study, the forces applied by AMS‐treated teeth and the control teeth seemed to be linked to the magnitude of the tested load masses. Particularly, higher forces were employed for the heavier load masses whereas the lower forces were employed for the lighter load masses.

In the current study, it was observed that AMS‐treated teeth demonstrated a significantly lower peak force and peak force rate in the output of force profile when compared to the control teeth, during the initial time‐segment. It is well recognised that AMS treatment is aimed to heal the post‐treatment apical periodontitis, restore the function of the teeth and improve the patient's quality of life.^19^, ^20^ While the AMS treatment is a less invasive procedure, the occurrence of pain and swelling after the treatment is not uncommon.^29^ Several studies on the gait of humans have suggested that older individuals tend to walk with a cautious gait due to fear of falling.^30^, ^31^ Therefore, it can be speculated that the presence of pre‐operative and /or post‐operative pain or symptoms in the AMS‐treated teeth could result in a ‘too cautious’ approach of the participant towards the applied forces during the unpredictable force control task. This ‘cautiousness’ against the applied forces in the AMS‐treated tooth perhaps eventually results in a lower peak force. Furthermore, even though the AMS treatment can repair the periapical tissue and the surrounding structures,^32^, ^33^ the alteration in the morphology of the root‐end after the AMS treatment could result in a wider surface area of the root‐end, rendering a higher possibility of re‐population of the nerve endings during the healing process and greater sensitivity to the force load. These potential reasons could explain the lower peak force and peak force rate of the AMS‐treated teeth during the given initial time‐segment. In neuroscience, the initial time‐segment is generally attributed to the exploration of the novel spatial relationships between the object, the body, and their interactions (i.e. exploratory stage).^34^ After the initial exploration, the individual generally shifts the focus to a more confirmatory stage and later to an anticipatory stage.^35^ Therefore, it was reasonable/hypothesised to assume that the confirmatory stage, that is, the later segment (holding force) in the current phase is more vulnerable to changes in the force control due to perturbation of sensory information as a result of resection of the root apex.

During the later time‐segment, the force profile output demonstrated no significant differences in the holding force or the coefficient of force variability between the AMS‐treated teeth and their controls. Indeed, the motor neurons in the later time‐segment are frequently used the saved/gathered information about the load masses during the initial time‐segment and consequently predict the required motor commands and expect the sensory consequences.^9^ This clarifies the similarity in the holding force and the force variability between the AMS‐treated teeth and the control teeth. Further, the force variability during the unpredictable force control task is a reflection of the noisy environment in the central nervous system that originates and propagates through the sensorimotor system.^36^ It has been suggested previously that at lower force output, fewer motor signals are generated, which leads to an increase in the force variability.^37^ In the present study, the force variability showed an inverse relationship to the increase of the load mass. This observation is in accordance with previous studies that showed an increased force variability with a lighter load than heavier load mass.^8^, ^9^, ^37^ Typically, the variability in the force was lesser with the heavier load masses and higher with the lighter load masses in both AMS‐treated teeth and the control teeth.

The current unpredictable force control task generated quite low forces during the initial and later time‐segments of the force profile. Previous studies in endodontics have assessed the occlusal bite force of the root canal‐treated teeth in comparison with the vital teeth.^20^, ^38^ Nonetheless, the results obtained from these studies seem to be inconclusive. While one study reported higher occlusal forces in the root canal‐treated teeth than the vital teeth,^38^ another study reported no significant differences in occlusal forces between the root canal‐treated teeth and the vital teeth.^20^ The variabilities in the results of the previous studies could be related to different methodological aspects. Unlike the current unpredictable force control task, the occlusal bite force method generates quite high forces, which can be affected by masticatory muscle activities.^39^, ^40^ Moreover, the quality of the root canal treatment and the treatment outcomes were not considered in the previous studies.^20^, ^38^ One of the main indicators for a successful endodontic treatment is the resolution of apical periodontitis.^41^ Although healing of the apical periodontitis necessitates an adequate root canal filling, a longer follow‐up period might be required for reconstruction of the periapical area.^42^ Failure to adjust these confounders may influence the accurate interpretations of the previous results. Further, it had been shown that a minimum of one‐year follow‐up is essential to determine the periapical healing after the AMS treatment.^24^, ^25^ All in all, the current pilot study followed a stringent inclusion criterion for the AMS‐treated teeth, presented by the healing of the apical periodontitis with a minimum of one‐year follow‐up. The current findings also corroborate our previous findings in a similar group of patients that showed no significant difference between the teeth treated with apical microsurgery and the control teeth in holding forces during a more standardised ‘hold and split’ behavioural task.^7^

Clinical studies often involve a number of methodological concerns, which should be addressed appropriately. One such methodological concern was the relatively small sample size, the large age range of the participants, and in particular inclusion of older individuals in the study. Since ageing has its effects on sensorimotor regulation and jaw function, this could be a confounding factor in the study.^43^ However, the paired design where each participant is his/her own control and the randomisation of both the order of the teeth (AMS/contralateral control) and the sequence of load (50/100/200/300) were randomised could be a positive attribute in the current study. Therefore, it is suggested that despite the concerns, the interindividual variability is eliminated and the intrinsic changes due to ageing are minimised. In addition, incisor teeth were selected over posterior teeth due to their higher sensitivity to the changes in the applied occlusal forces.^7^ Despite this, the findings of the study can perhaps not be reasonably extrapolated to the posterior teeth due to the presence of multiple roots in the posterior teeth. It is also appropriate to acknowledge that the force profiles are related to the electromyographic activity of the jaw muscles; therefore, future studies may also include measurement of the electromyographic activity of the muscles involved in the other behavioural/ force control tasks. Nonetheless, future studies should also be targeted towards other illuminating approaches including brain imaging and quantitative sensory testing, which could be useful tools in evaluating the force regulation of the teeth and the subjective sensory experience of the individuals during the unpredictable force control task.

## 5 CONCLUSIONS

Overall, the AMS‐treated incisors showed differences in peak force and force rate compared with the control teeth, during the initial time‐segment (explorative stage) of the force profile. However, contradictory to our hypothesis there was no major difference in the holding force and the coefficient variability of the holding force between the AMS‐treated incisors and the control teeth in the later time‐segment (confirmatory stage) of the force profile. Therefore, the force regulation of AMS‐treated incisor teeth is not significantly different from teeth with normal root apices. These results may imply that the AMS treatment does not jeopardise the force regulation of the incisor teeth despite the removal of the root‐end. It can also be acknowledged that AMS treatment plays a crucial role in maintaining the tooth function besides the healing of post‐treatment apical periodontitis.

## CONFLICT OF INTEREST

The authors declare that they do not have any conflict of interest related to this study.

## AUTHOR CONTRIBUTIONS

**Khaled Al‐Manei** designed and carried out the experiments, performed analyses, interpreted the data and results and wrote the original draft. **Nabeel Almotairy** carried out the experiment, performed analyses and edited the manuscript. **Kholod Khalil Al‐Manei** designed the experiments, interpreted the results and edited the manuscript. **Anastasios Grigoriadis** conceptualised the project, supervised the project and edited the manuscript. **Abhishek Kumar** conceptualised the project, methodology, critical feedback on the results and edited the manuscript. All authors have read and agreed to publish this version of the manuscript.

## Data Availability

The data used to support the findings of this study are included within the article.
